# Mission Command in the Age of Network-Enabled Operations: Social Network Analysis of Information Sharing and Situation Awareness

**DOI:** 10.3389/fpsyg.2016.00937

**Published:** 2016-06-22

**Authors:** Norbou Buchler, Sean M. Fitzhugh, Laura R. Marusich, Diane M. Ungvarsky, Christian Lebiere, Cleotilde Gonzalez

**Affiliations:** ^1^U.S. Army Research LaboratoryAberdeen Proving Ground, MD, USA; ^2^Department of Social and Decision Sciences, Carnegie Mellon UniversityPittsburgh, PA, USA

**Keywords:** network organization, sociotechnical system, Pareto principle, communication exponential random graph model, homophily, degree distribution, training effectiveness

## Abstract

A common assumption in organizations is that information sharing improves situation awareness and ultimately organizational effectiveness. The sheer volume and rapid pace of information and communications received and readily accessible through computer networks, however, can overwhelm individuals, resulting in data overload from a combination of diverse data sources, multiple data formats, and large data volumes. The current conceptual framework of network enabled operations (NEO) posits that robust networking and information sharing act as a positive feedback loop resulting in greater situation awareness and mission effectiveness in military operations (Alberts and Garstka, 2004). We test this assumption in a large-scale, 2-week military training exercise. We conducted a social network analysis of email communications among the multi-echelon Mission Command staff (one Division and two sub-ordinate Brigades) and assessed the situational awareness of every individual. Results from our exponential random graph models challenge the aforementioned assumption, as increased email output was associated with lower individual situation awareness. It emerged that higher situation awareness was associated with a lower probability of out-ties, so that broadly sending many messages decreased the likelihood of attaining situation awareness. This challenges the hypothesis that increased information sharing improves situation awareness, at least for those doing the bulk of the sharing. In addition, we observed two trends that reflect a compartmentalizing of networked information sharing as email links were more commonly formed among members of the command staff with both similar functions and levels of situation awareness, than between two individuals with dissimilar functions and levels of situation awareness; both those findings can be interpreted to reflect effects of homophily. Our results have major implications that challenge the current conceptual framework of NEO. In addition, the information sharing network was largely imbalanced and dominated by a few key individuals so that most individuals in the network have very few email connections, but a small number of individuals have very many connections. These results highlight several major growing pains for networked organizations and military organizations in particular.

## Introduction

Advances in information and network technology continue to transform the way human organizations communicate and operate. This is evident as networked organizations are at the core of the political, military, economic, and social fabric of the twenty-first century (Castells, 2009). The same technological advances that have given rise to networked forms of organization also facilitate their study. For example, larger and larger volumes of data that characterize our “digital behaviors,” including communication and collaboration, are increasingly collected by companies, governments, and researchers alike (Navaroli and Smyth, 2015). Using this digital behavior data, organizations can be characterized as social networks with nodes representing individuals and links representing the interactions between them. Many such networks are inherently complex in the sense that their structure is irregular, task- and context-specific, and dynamically evolving in time.

Over the past decade, the social sciences have seen rapid growth in research and understanding of the structure of real-world complex networks (Borgatti et al., 2009). However, the effects that operating within such complex networks have upon individual macro-cognitive processes is not well understood (Klein et al., 2003). Organizations can confer considerable advantages to information sharing as the number of potential collaborations may be virtually limitless, as is the availability of information. There are however, some potential downsides as well, as the resulting deluge of information (Gleick, 2012) can quickly overwhelm human cognitive capabilities. Understanding the relationship between network structure, human collaboration, and cognitive work processes within real organizations is a critical challenge. This is especially true in command and control domains, such as military operations, emergency response, managing safety critical systems, air traffic control, computer network defense service providers, and others. In all these naturalistic domains information from various sources and of varying quality must be quickly assimilated and shared among distributed team members to make critical decisions with potentially significant consequences.

A prevalent perspective within these domains is that increased networking capabilities lead to greater information sharing and availability of information which ultimately results in improved collaboration, organizational efficiency, and better situation awareness (SA). We explore this assumption, investigating macro-cognitive processes using data collected in a large-scale exercise of military network level operations. We focus on the relationship between information sharing and SA within a real-world networked organization.

### Network enabled operations and information fusion

The tenets of network-enabled operations (NEO; Alberts and Garstka, 2004) provide an influential conceptual framework for understanding how increased networking affects human collaboration and organizational performance within the military domain. This framework posits that communication and information sharing act as a positive feedback loop with increased information sharing resulting in greater situation awareness and mission effectiveness in military operations. From a policy perspective, enhancing information sharing within and across organizations has been and is a major priority for investment by the United States government including the Department of Defense (Alberts et al., 1999; Alberts and Garstka, 2004), Federal Emergency Management Agency, Department of Homeland Security (Department of Homeland Security, 2013), and the Federal Aviation Administration (2014). As information sharing is increasingly promoted within NEO, it becomes critical to explore and understand the relationships between information sharing, cognition, and situation awareness among the staff in these complex operational environments.

The positive effects of increased information sharing upon SA can be greatly diminished if individuals reach a state of information overload. A major tenet of the Office of the Secretary of Defense's “data-to-decision” (D2D) initiative (Swan and Hennig, 2012), and a primary challenge for military commanders and their staff is to shorten the cycle time and improve the processes of synthesizing data to information and into knowledge to support decision-making and action. Organizational performance and effectiveness are curtailed by failures or bottlenecks at any step in this D2D sequence. Effectively managing the entire process requires broad collaboration and flexibility in supporting multiple information and decision requirements. In networked organizations, however, the sheer volume and rapid pace of information and communications received and readily accessible from diverse sources and in multiple formats can quickly overwhelm individuals in the D2D pipeline. Well-designed automation and decision-support tools can provide some assistance in the D2D cycle; however, the volume of data flowing through large organizational networks is often beyond the ability of current software tools to capture, curate, and store (Salimi and Vita, 2006; White, 2012) or to process the data within a tolerable time frame (Snijders et al., 2012).

A critical process of the D2D pipeline is that of information fusion. Software tools and automation currently lack the capabilities to synthesize information in an adaptive, highly context-aware manner, which necessitates human involvement and considerable cognitive resources (Blasch et al., 2011). Many contextual factors affect the human ability to rapidly synthesize information into a coherent understanding of the current situation, including information volume, quality, and modality, the general level of risk and time-pressure in the environment, and factors operating at the level of the individual decision-maker, including cognitive load, fatigue, level of expertise, and personality traits such as need for closure and need for cognition. The concept of cognitive information fusion (Blasch et al., 2012) emphasizes the necessity and strength of the human element in order to achieve a high-level, contextual understanding of a given situation. Data fusion is a term typically used to describe computational frameworks for constructing a comprehensive, data aggregation system that processes information to support user decision-requirements (Klein, 2004), whereas cognitive information fusion explicitly emphasizes the need for human cognition and staff collaborations to integrate and rapidly make sense of these data streams that are distributed across space and time. The outcome of proficient cognitive information fusion is high situation awareness, which we describe in detail below.

### Situation awareness in NEO

Situation Awareness (SA) is defined as “the perception of the elements in the environment within a volume of time and space, the comprehension of their meaning and the projection of their status in the near future” (Endsley, 1995, p. 36). SA is a well-known concept in a variety of domains that require cognitive information fusion, including military operations (Endsley, 2000; Matthews et al., 2004), aviation (Kaber et al., 2002; Keller et al., 2004), air traffic control (Endsley and Kiris, 1995; Endsley and Smolensky, 1998; Hauss and Eyferth, 2003), transportation (Zheng et al., 2004) and many others. The three-level model of SA proposed by Endsley (1995) is perhaps the most common model of SA (other models include those discussed in Smith and Hancock, 1995 and Bedny and Meister, 1999). Endsley's model depicts SA as an essential input into human decision-making cycles that is composed of three hierarchical levels: (level 1) the perception of the elements in the environment (level 2), the comprehension of their meaning, and (level 3) the projection of their future status. In the current work we use SA as a measure of an individual's success at performing cognitive information fusion to comprehensively understand the current status of events transpiring on a simulated battlefield.

At the cognitive or nodal level, the relationship between information, situation awareness, and task effectiveness has been extensively investigated in a number of ways including carefully controlled laboratory behavioral experiments. For example, Gonzalez and Wimisberg (2007) demonstrated that practice effectively improved information processing, the attainment of SA, and performance on dynamic decision tasks. Further, training reduced the relationship between individual cognitive abilities and SA to suggest that the cognitive demands of maintaining SA are reduced with practice. Also, a recent laboratory-based study examining human performance on simulated command and control tasks found that, contrary to expectations, increasing the volume of task-relevant information did not improve task performance, but instead reduced self-reported SA, leading to poorer task performance (Marusich et al., 2016). These results suggest that increasing the volume of information, even when it is accurate and task-relevant, is not necessarily beneficial to decision-making performance and may be detrimental to SA among team members. Military operations, however, are inherently complex human endeavors involving macro-cognitive processes that cannot be fully recreated or studied in the laboratory (Klein et al., 2003). As such, it is unclear whether these laboratory findings regarding the effects of practice and increasing volumes of information on SA also manifest themselves in naturalistic settings. As commanders and their staffs collectively face difficult, stressful, and dynamic challenges in managing battlefield operations, we need to determine the effects of information sharing, cognition, and training on their SA in more complex, real-world environments.

Warfare is chaotic and extremely complicated. Resolving the attendant ambiguity on the battlefield is both a cognitive and collaborative challenge of the first order. In these situations, human integration of networked information among the mission command staff is essential to successful military operations. A possible way to reduce the potentially detrimental effects of information overload is to distribute information processing tasks across the network—allowing separate people to process and act upon different sets of information (see, Kozlowski et al., 1999; Salas et al., 2008). In this case, a broad distribution of information and SA is essential for NEO. However, such distribution may also create added communication and coordination costs as well as additional dependencies, requiring each person in the network to maintain awareness of the dynamic situation and rely on the performance of others. Some research in military-relevant field exercises demonstrates a significant relationship between SA and the participants' awareness of the information in the central nodes of a team (Saner et al., 2009). This result suggests that SA is centralized and not broadly disseminated across the networked organization and that a person's role and position within an organization affects and potentially limits the level of shared SA that can be achieved. Our study scales up the results of these studies at the level of the individual and small teams to examine organizational network levels of performance.

The focus of our research is to examine and characterize the relationship between information sharing behavior and the distribution of SA in a real-world networked military organization. We examine how collaboration and information sharing among a large, networked mission command staff affects the attainment and distribution of individual SA across a 2-week real-world military exercise. Specifically, we construct network graphs from the record of staff communications throughout the exercise, and assess how the structure of these graphs relates to the SA of individuals within the network, as well as how this relationship evolves over the course of the exercise. Our results characterize the relations between volume of information, SA and performance and have major implications for training and systems design in NEO domains. Next, we describe this training event and our data collection and analysis.

## Mission command training exercise event

The Mission Command Battle Laboratory at Fort Leavenworth, Kansas conducted a training event exercise focused primarily on the mission command operations of staff composed of a Division headquarters (*n* = 46) and two subordinate Brigade headquarters (*n* = 21, *n* = 23). Additional units and staff at echelons above and below the Division and Brigades participated in the training event exercise, with the size of the networked organization in excess of 200 (*n* = 213). The network architecture and digitized nature of the event allowed examination of staff communications in a distributed, network-enabled environment. Below we describe the defining characteristics of this military organization, and the nature of the tasks they were required to complete.

### Defining characteristics of the military organization

The participants were active duty (and in a few cases retired): Soldiers and officers with operational staff experience who were assigned to differentiated, well-specified, and inter-dependent roles. Several staffs at different echelons participated, including a functional slide of a Division operations center and the staffs of a U.S. Heavy Brigade Combat Teams (mechanized) and a U.K. Coalition Brigade Combat Team. The units operated in a distributed fashion (U.S. units at Fort Leavenworth and the U.K. unit at the Land Warfare Centre in Warminster) over a communication network using specialized military command and control hardware and software. Within each unit, staff members carried out the duties of nine different functional cells. These cells included Command, Maneuver, Intelligence, Fires, Civil Affairs, Signal, Sustainment, Protection, and Liaison. Individual responses and responsibilities to a given scenario event in the training exercise depended upon adherence to established workflows and standard operating procedures both within the unit and functional cell.

Several additional small units and staffs were presented in the exercise, including high command elements of an International Joint Command, as well as a Civil Military Operations Center to facilitate coordination of joint, interagency (e.g., Department of State, United States Agency for International Development), intergovernmental, and multinational efforts. In addition, a third Infantry Brigade Combat Team was notionally represented; however, their area of operations was quiet and not fully exercised by scenario events. At the lowest level, a number of key role positions were staffed to represent Battalion level units in Army Aviation, Engineering, Military Police, and Sustainment (i.e., Counter Improvised Explosive Device). We used an electronic survey instrument to collect SA information from the Division, Heavy Brigade Combat Team, and the Coalition Brigade Combat Team, as these groups and their interoperability were the primary focus of the exercise. The high command elements, the Infantry Brigade Combat Team, and Battalion level units did not receive the electronic survey.

The military organization was staffed and convened specifically to execute and accomplish a particular 2-week long training mission. They worked interdependently and engaged in collaborative decision-making for mission planning and execution. The organization functioned as a *purposive social system*, where members are readily identifiable to each other by role and work interdependently to accomplish one or more collective objectives (Hackman and Katz, 2010). The responsibility for performing the various tasks and sub-tasks necessary for mission success is divided and assigned among the staff.

### Defining characteristics of the tasks

The training scenario in a military exercise generates many overlapping series of event-driven tasks, the resolution of which requires a high degree of coordination among the participating command and control staff. Researchers have long pointed out that the nature of a task has a great influence on the steps and processes a group uses to perform the work (e.g., Roby and Lanzetta, 1958; McGrath and Kravitz, 1982). The tasks of groups in the military domain considered here have four distinguishing features:

1. *Specific Presenting Problems:* The military command and control staff is tasked with addressing specific problems that occur in the unit's area of operations. The military staff organization must monitor key events and successfully plan and coordinate an effective response, given limited resources. The presenting problems may be kinetic events, such as responding to an improvised explosive device, or civil-military in nature, such as responding to a civil demonstration or safeguarding polling sites and maintaining a chain of custody in the transfer of voting ballots. At other times, the presenting problem may be a time-sensitive intelligence report of enemy activity that needs to be analyzed and corroborated. At any given time, the organization must coordinate a response to many such presenting problems.
2. *Adherence to specific tactics, techniques, and procedures:* The groups adhere to formalized military work routines and processes that are known in advance and involve delegation of specific work responsibilities to various sub-groups and individual staff members.
3. *Addressed immediately:* The group operates in an urgent, time-sensitive work environment and is required to immediately coordinate responses to work events that may have adverse cascading effects if not addressed in a timely manner.
4. *Results in collaborative work products that need to be coordinated and disseminated:* The group is expected to construct specific, detailed material products that will exist independently of the group process or the individual members themselves. For instance, the Commander and his command elements require regular reports from the staff in order to achieve situational awareness of the battlefield environment. The work processes themselves and the dissemination of both intermediate and final work products occur across the communication network as observable behaviors over time.

### Data collected

#### Communications network

**Figure 1:**
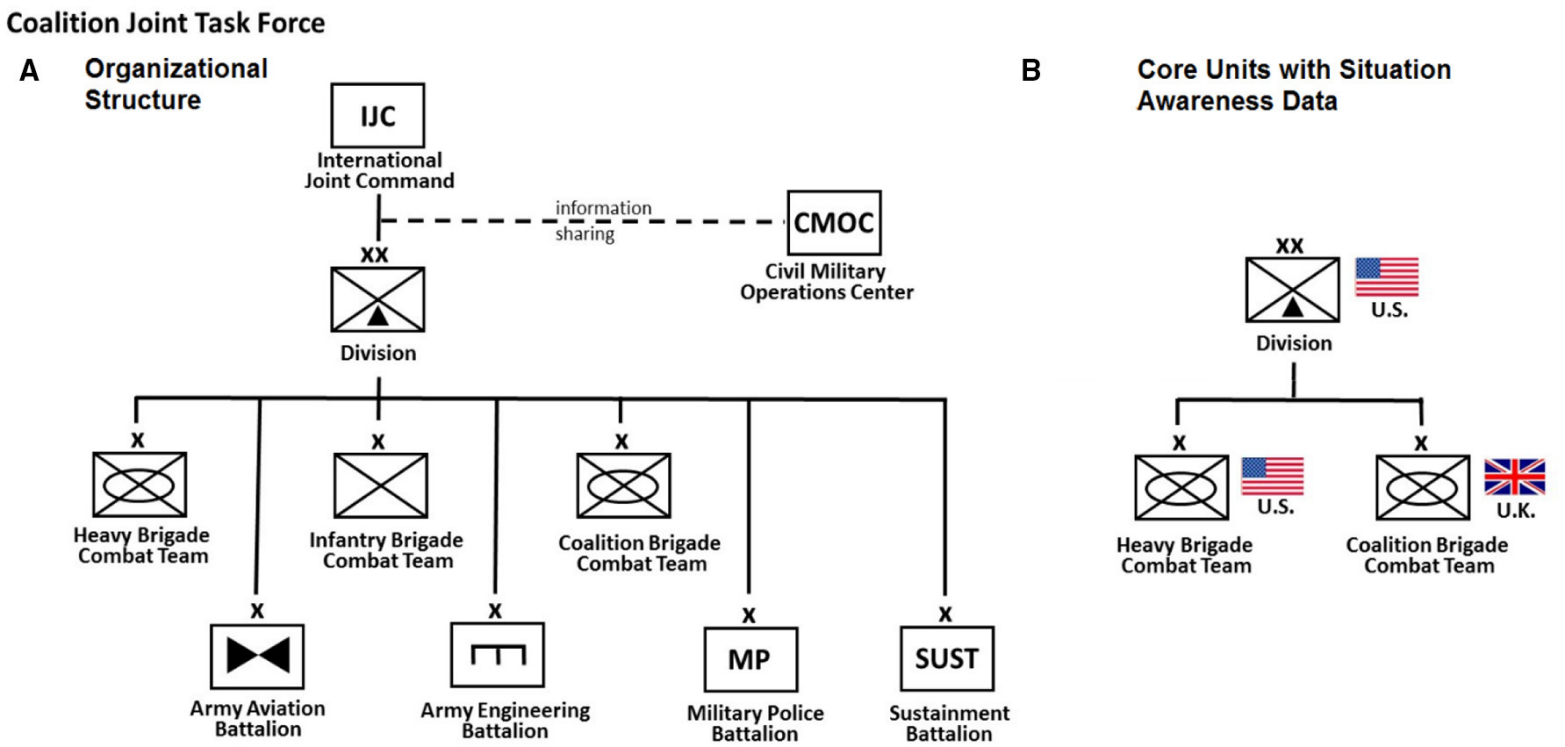
**(A)** The organizational structure of the Coalition Joint Task Force during the 2-week military training exercise event held at the Mission Command Battle Laboratory (Fort Leavenworth, Kansas). The networked organization spans multiple echelons from Joint Command to Division to Brigade to support-Battalions. Communications were collected for the entire Coalition Joint Task Force organization. **(B)** The core units exercised during the training event consisted of the Mission Command staff of a U.S. Division and two participating sub-ordinate Brigades, a U.S. Heavy Brigade Combat Team and a U.K. Coalition Brigade Combat Team. Individual situation awareness data was collected using the SAGAT methodology from the participating staff of these three core units.

Telephone and email were two primary methods of direct communication between staff members during the exercise. For each email message sent and phone call made in our dataset, three pieces of information were automatically logged electronically: the sender, the receiver, and the time of the communication's initiation. The resulting full communications network consisted of: (a) an email network of 213 mission command staff members and 19168 correspondences, and (b) a telephone network of 3191 calls between 132 mission command staff members. The survey methodology, however, was only applied to the core units of the Coalition Joint Task Force organization. Thus, a subset of the email communications network (see Figure 1, right panel) is subsequently visualized and used for our statistical model analysis of information and situation awareness. The telephone network was sparse and did not fully represent all the members of the core staff and thus not subjected to statistical model analysis.

#### Situation awareness global assessment technique (SAGAT)

A valid and reliable method for assessing SA is essential for understanding whether information sharing behavior improves the SA of the personnel involved in the networked organization. Techniques such as the Situation Awareness Global Assessment Technique (SAGAT; Endsley, 1995) and the Situation Awareness Rating Technique (SART; Taylor, 1990) have been applied in a number of organizational settings including military operations (Salmon et al., 2006), medical care environments (Wright et al., 2004), robot control (Chen et al., 2011), and industrial processes (Patrick et al., 2007).

In our electronic survey, we used the SAGAT, a widely used and validated SA measure (Endsley, 2000; Sonnenwald and Pierce, 2000) that makes use of a *pop quiz* memory probe technique to immediately present a set of questions to an individual regarding the state of their current task environment. The SAGAT methodology freezes the event to assess individual SA using targeted sets of online queries (multi-item quiz). The SAGAT methodology was developed and administered twice daily using online queries; at two predetermined times each day, an electronic questionnaire popped up on the computer monitor of each Mission Command staff member. After completing the questionnaire and submitting their responses, the Mission Command training exercise resumed.

Implementing the SAGAT requires advanced knowledge of the events so that a targeted set of queries can be developed and administered to the participating Mission Command staff. Each set of SA questions was determined in consultation with the lead mission planner coordinating the training exercise event, who determined the best times to administer the SA queries. Significant mission events that were expected to occur prior to the query time were identified and questions that would assess SA on these relevant events were selected. The questions were developed from an SA requirements analysis conducted for various Army Mission Command staff positions using goal-directed task analysis methodology (see Bolstad and Endsley, 2003). During the event, subject-matter experts tailored the queries to the unfolding events and relevant aspects of mission in the area of operations for each Unit: US Division, UK Brigade, and US Brigade. The SA queries were broadly applicable, and not tailored to each role. Everyone received the same SAGAT queries but the answers were unit-specific. For example, the answers to the query “In your sector, which of the following CIVILIAN ACTIVITIES are currently occurring?” would be different for the US Brigade and the UK Brigade based on what was happening in their area of operations. Ground truth was determined based on tracking events in the simulation and feedback from subject-matter experts controlling the scenario-based exercise.

Each individual SA questionnaire included on average eight items from a total pool of 33 general queries. Unanswered questions were scored as incorrect. Questions were scored based on the participant's base unit. The data was collected by a contracted partner, SA Technologies Inc., to the Mission Command Battle Laboratory and provided to us in the aggregate for week 1 and week 2 of the exercise event. A sample set of queries is given in Table 1.

**Table 1:** **Sample 19-item quiz administered to mission command unit using SAGAT methodology**.

1.	At this time, the MOST significant CIVILIAN event involves which of the following?
2.	At what LEVEL have CYBER ATTACKS been directed against Coalition operations in the last 4 h?
3.	Do you currently have troops in contact?
4.	Has a Commander's Critical Information Requirement been reported in the LAST 4 h?
5.	Have you received ACTIONABLE INTEL in the last 4 h REGARDING High Value Targets in your Area of Operations?
6.	How LONG has it been since the last MEDEVAC in your Area of Operations?
7.	In which portion of the Area of Operations was the LAST CALL for FIRES?
8.	In your sector, which of the following CIVILIAN ACTIVITES are currently occurring?
9.	The LAST REQUEST in your Area of Operations from CIVILIAN leaders was for which of the following?
10.	The MOST RECENT DETAINEES in your sector were engaged in which of the following BEFORE CAPTURE?
11.	What is the NATURE of the most recent REQUEST from COALITION/HOST nation partners?
12.	What type of targets will Counter Coalition Forces attack within the NEXT 2 h?
13.	What was the COALITION RESPONSE to the last attack in your sector?
14.	What was the NATURE of the last incident reported?
15.	What WEAPONS did the Counter Coalition Forces employ in the LAST attack in YOUR SECTOR?
16.	Which of the following best describes the TARGET of the last Counter Coalition Forces attack in your sector?
17.	Which of the following describe the OUTCOME of the last attack in your sector?
18.	Which of the following have been INCORRECT or EXAGGERATED in media reports in the last 4 h?
19.	Which of these INFRASTRUCTURE SERVICES are disrupted in your Area of Operations?

## Results

### Social network visualization

**Figure 2:**
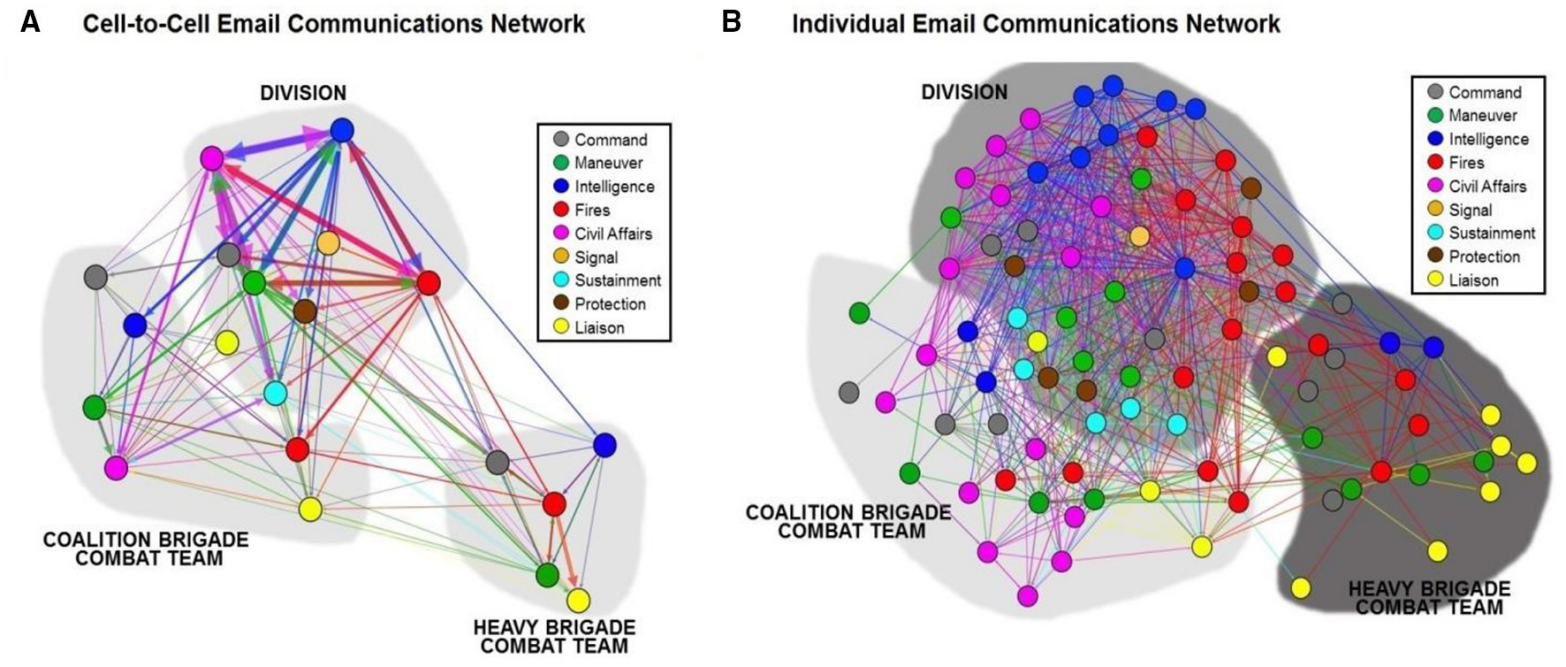
**Network visualization of email communications between the Mission Command staff across a 2-week training exercise event encompassing two echelons of Command—a Division and two-subordinate Brigades**. Email communications are aggregated at the cell level to reveal functional cell-to-cell correspondences **(A)** and disaggregated at individual node level **(B)**. Node color indicates functional cell assignments for all members of the Mission Command staff, which are specified in the legend. The color and thickness of the lines denote the functional cell of the sender and message volume.

A network is defined as a set of nodes and the connections between them, called edges (undirected) or arcs (directed). In our organizational network of military command staff, the social collaborations are represented as directed email connections between individual nodes. The strength of a connection—number of email correspondences between nodes—is represented by the thickness of the line. At aggregate levels of analysis, the nodes can be grouped into units and cells to understand functional information flows. There were 45 individuals in Division roles, 23 in U.K. brigade roles, and 21 in U.S. Brigade roles, for a total of 89 nodes that were used in both our network visualization and subsequent statistical model and analysis. The pattern of email communications highlights the complex interdependencies and information sharing among the Mission Command staff (Figure 2B) and the diverse information flows between functional cells. The layout of the network visualization was produced using Gephi—an open-source network analysis and visualization software package—and is energized to minimize the overall variation in line length using the Force Atlas (Jacomy, 2009) algorithm. This algorithm effectively centralizes the most highly-connected nodes and pushes the least connected nodes to the periphery. Our levels of analysis extend from the unit-level (e.g., Division, Brigade, and Battalion) to function-cell (Command, Maneuver, Intelligence, etc.) all the way down to characterizing individual staff members. The network visualization highlights the sheer complexity of current information sharing environments to achieve coordination and unity of effort among the Mission Command staff.

### Imbalanced information sharing

**Figure 3:**
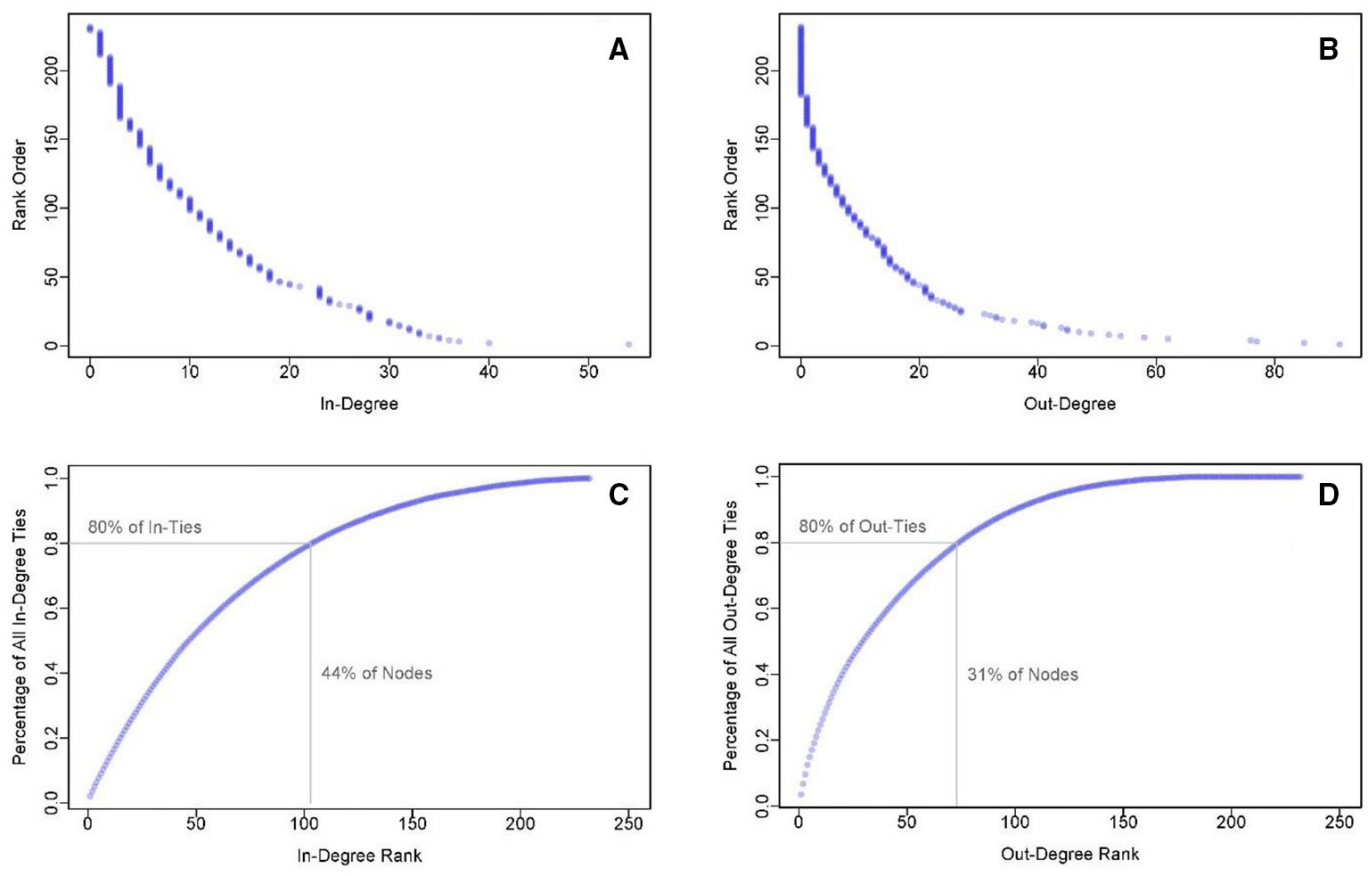
**In-degree (A) and out-degree (B) cumulative distribution functions for the full email communications network**. Such heavy-tailed distributions are common in complex networks. The dominance of some members of the Mission Command staff is evident when expressed as a percentage of all ties (email connections) for in-degree **(C)** and out-degree **(D)**. The inserted lines show the percentage of nodes that subsume 80% of the in-ties or out-ties.

The distribution of email communications among the command staff is represented by in-degree and out-degree. The in-degree of a node is the number of individuals who send messages to that node. Conversely, the out-degree is the number of individuals who receive messages from that node. In our observed mission command network, a fundamental asymmetry exists in the degree that distribution of information flows among staff email collaborations. A few key individuals dominate information sharing among the staff. This is apparent in the cumulative distributions of in-degree and out-degree of email correspondences (see Figures 3A,B). These plots show the number of individuals with degree greater than or equal to a specified value. Most individuals in the network have very few connections, but a small number of individuals have many connections. Steeper drop-offs in these plots correspond to greater asymmetry in the degree distribution. The dominance of key members of the Mission Command staff conforms to a general network property of complex systems. The degree distributions of real-world networks are typically skewed and non-normal (i.e., non-Gaussian) with heavy tails (Barabási et al., 1999; Strogatz, 2001). Heavy-tailed distributions are so pervasive in real-world networks—turning up again and again in a wide variety of both natural and social phenomena, from earthquakes and floods to wealth, talent, and Internet behavior (West, 2012) that in organizational settings this phenomenon is known as Pareto's Law of the vital few (20%) and the trivial many (80%). At the macro level, Pareto (1897) first described imbalances in the wealth distribution of western countries such that 20% of the people owned 80% of the wealth. The seminal importance of this pioneering work is noted by West (2012, p. 78), who describes Pareto as “the first to have the modern vision of society as a network of reciprocal and mutually interdependent entities.”

In our email communication network, key individuals at the tail of the degree distribution were found to dominate collaborations. The steeper drop-off of Figure 3B, as well as the more extended tail indicates that this was even more evident in the out-degree distribution than the in-degree distribution. We examine these degree imbalances in terms of the Pareto phenomenon (see Figures 3C,D). Degree rank is plotted on the x-axis, with 1 being the individual with the highest degree, and the percentage of all in-degree connections in the network belonging to that individual is plotted on the y-axis. Here again, a steeper curve indicates a greater imbalance in the degree distribution. We mark on the curves the points denoting how many individuals are responsible for 80% of the ties. In the in-degree distribution, 44% of the staff were responsible for 80% of the in-ties. In the out-degree distribution, only 31% of the nodes were responsible for 80% of the out-ties, nearing the classic Pareto distribution. Ultimately, this is interpretable as the implicit imbalance and pervasiveness of heavy-tailed distributions in complex networks.

### Exponential random graph statistical model

Exponential-family random graph models (ERGMs) are a family of statistical models widely used for inferential analysis of social network data (Hunter et al., 2008). Observed networks are standalone instances of many possible realizations of a given network. To support statistical inference about the structure of a given network, an ERGM compares the similarity of the current observed network to the set of all possible alternative configurations. This allows us to establish a statistical baseline to infer the likelihood that the network could have expressed the observed structural characteristics at random. The ERGM models described below give the probability of observing a particular structural edge—an email connection—as a function of the model parameters, which are based on a variety of statistics from the network. The coefficients are not unlike those in a logistic regression, and can be interpreted as their effect on the log-odds of observing a given edge. In the email network, for example, the log-odds of observing an edge that reciprocates another edge is significantly higher than observing an edge that does not reciprocate an edge.

**Figure 4:**
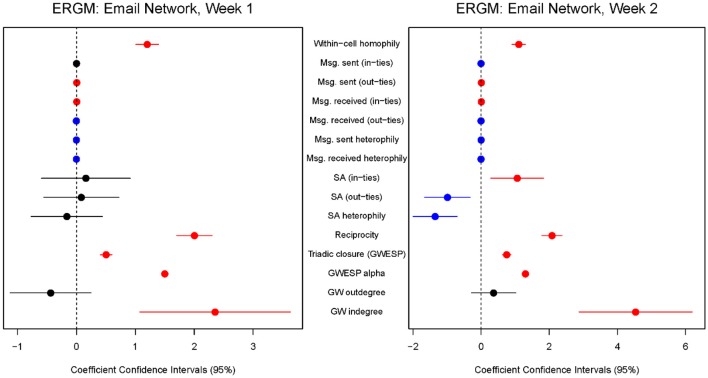
**Exponential random graph statistical models of the email communication network during week 1 (left panel) and week 2 (right panel) of the Mission Command training event exercise**. The models describe the probability of observing any given edge as a function of the coefficients (log odds) in the statistical model. Results that are positive and significant are colored red, results that are negative and significant are colored blue, and results that are not statistically significant are colored black. The circle represents the statistical coefficient while the lines represent the 95% confidence interval for the coefficient. Note that given the large volume of messages some nodes have very small and significant effects even though they appear to be sitting on the 0 mark.

Using the ergm package in R (Handcock et al., 2016), we fit separate ERGM models to Week 1 and Week 2 of the exercise (Appendix). The model coefficients for each week are plotted in Figure 4. Results that are positive and statistically significant are colored red, results that are negative and significant are colored blue, and results that are not statistically significant are shaded black. The circle represents the value of the coefficient and the lines represent the accompanying 95% confidence interval. Due to the sheer number of communications in our dataset, some model coefficients have very small but significant effects even though they appear to sit on the 0 mark. We describe the effective terms of the statistical model in detail below.

#### Robust information sharing environment

Across both weeks, we find strong positive effects for within-cell homophily, reciprocity, triadic closure, and indegree. Homophily refers to the observation that networks often foster connections based on similarity (McPherson et al., 2001); in our case, defined as other individuals of the same functional cell (see Figure 2). These functional cells are well-defined and known according to the general staff system (Department of the Army Headquarters, 2015) and include: command, maneuver, intelligence, fires, civil affairs, signal, sustainment, protection, and liaison. During both weeks the model demonstrates a propensity for within-cell ties in the communication network. Reciprocity in directed email communications between two individuals (dyads) refers to the likelihood of mutual connections or email exchanges between them. We found a high propensity for reciprocity of email exchanges between individuals. That is, in a directed graph, if individual A sends email to individual B there is a strong likelihood that B also sends an email to A. More elaborate social structures arise when considering three individuals (triads) since a much wider set of interactions is possible among them. Triadic closure refers to a property of social networks that if relations exist between two pairs of individuals (A-B and A-C), then there is a strong likelihood of a tie (B-C) that completes the triangle of relations. Both reciprocity and triadic closure are common features of social networks (Scott, 2012). The model terms indegree, outdegree, and triadic closure were geometrically weighted to control for preferential attachment effects so that each additional shared partner has a declining positive impact on the probability of one or two persons forming a tie. This has been shown to work well in overcoming model degeneracy effects (i.e., bimodality) and in producing generalizable models that accommodate source and sink effects (see Hunter and Handcock, 2006; Hunter, 2007).

Our model also examines the association between tie formation and the number of messages sent or received, independent of degree. Across both weeks we find a positive, significant effect for the number of sent messages and out-degree [Msg. sent (out-ties)] and also between the number of received messages and in-degree [Msg. received (in-ties)]. As the volume of messages sent or received increases, so does the number of channels through which the individual sends or receives those messages. That is, rather than continuing to direct messages to a single partner or small set of partners, an individual who sends many messages is likely to send those messages to a large population of alters. The same is true for incoming messages. For a separate treatment of this dataset, see Marusich and Buchler (2016) for a detailed account and model of the overall email communication time series (by volume) and how it relates to an external work variable—the occurrence of significant simulated scenario events during the training event exercise.

#### Information sharing and decreased situation awareness

Our central hypothesis is based on the tenets of NEO. Alberts and Garstka (2004) posit that increased information sharing in an organization improves situation awareness. The model coefficients for a link between situation awareness and in-degree [*SA (in-ties)*] or out-degree [*SA (out-ties)*] examine whether nodes with higher or lower situation awareness are more or less likely to send or receive ties. For Week 1, we obtained null results: the statistical coefficients were not significant as tie formation (number of in-ties and out-ties) was not associated with higher (or lower) levels of situation awareness among the mission command staff. In Week 2, however, we do find a relationship between SA and the propensity to receive and form network ties. Higher SA was associated with a higher tendency to form in-ties and a lower tendency to form out-ties. This challenges the hypothesis that increased information sharing improves situation awareness. The model coefficient for email in-ties and situation awareness was positive and significant, which indicates that those with high SA were more likely to be the recipients of ties. Receiving email (in-ties) implies a requirement for information, suggesting involvement in an organizational work process. On the other hand, sending email (out-ties) can be more material as it more directly advances an organizational work process especially if the information is processed and enhanced (value-added) and not just passed along. In other words, sending email is by definition an active process whereas receiving email is a passive process. Situation awareness is usually associated with an active process of constructing a mental model of the current events (Endsley, 2000), and thus should be associated with active work processes such as processing and sending email.

A plausible explanation of these results is that lower situation awareness is associated with work demands. The implication is that sending email demands attentional resources from the user and thus detracts from their overall situation awareness due to multi-tasking demands—in the same way that chatting with a passenger might distract a driver from paying attention to the route. An alternative explanation is that processing and sending email is associated with addressing specific challenges and fashioning work products, so that attention is not broadly allocated and instead tightly constrained and focused intently on processing a subset of features and events in the battlespace. A plausible explanation for the in-tie effect is that people with greater knowledge are likely to be tapped as potential sources of information and expertise, per transactive memory theory (Contractor and Monge, 2002; Borgatti and Cross, 2003), a rich-get-richer effect. Broadening the number of email out-ties was associated with lower situation awareness, perhaps because of the complexity of the operational environment outpaces human cognitive capabilities. In our broadly collaborative and information-rich Mission Command network, the accumulation of information can occur quite rapidly. In such cases, it can be difficult and time-consuming for the human operator to process relevant information and support workflows due to overwhelming volume of information and variety of different sources (email, chatrooms, maps with graphical overlays, imagery, video). An alternative explanation may be that those with higher SA did not find it necessary to reach out to others to obtain mission-critical information, with more in-ties they already had a firm grasp of their operating environment. Given the limits of causal inference, it is also possible that individuals with lower SA may send out more requests for information; disambiguating the direction of the effect requires an analysis of email content, which we do not have.

We note the development of an additional communication pattern that was cemented by week 2, according to the model coefficients. During the second week, we found positive effects for sending messages and establishing additional ties, in addition to receiving messages and receiving additional ties. We found that individuals with more incoming ties were less likely to *send* larger volumes of emails, while those with more outgoing ties less likely to *receive* more messages. Using the standard terminology of the network information flow perspective (Zachary, 1977; Ahlswede et al., 2000), taken together these four effects suggest that certain individuals increasingly act as *sources* or *sinks* of information in the networked organization. That is, they acted as either broadcasters or attractors of information. This reinforces the primacy of our earlier result that information is not shared equally in the network with Pareto-type imbalances to the in-degree and out-degree distributions.

#### Homophily in email communications

Homophily effects offer further insight into the pattern of SA that we observe in the network. During the second week we find a significant, negative effect for SA heterophily. That is, individuals with larger differences in SA are less likely to form ties with one another (i.e., lower SA individuals tend to communicate among themselves and higher SA individuals tend to communicate among themselves). This is another emergent property of the network, as we did not observe this pattern during week 1. Over time the network appears to become stratified with respect to SA. This could be the result of deliberate action—individuals with higher SA may reach out to others with high SA while avoiding those with lower SA—or this may be an outcome of the structural configuration of the network—those with access to information that enhances SA were unable to diffuse that information to parts of the network and, as a result, SA declined among those subgroups. The stratification of SA in this network is problematic for organizational performance and this problem deserves further attention both to advance organizational research and improve the effectiveness of military performance during training exercises. One possibility is that the organization is essentially divided into information processors whose job it is to understand the situation and who receive and send an inordinate number of email messages (and have high SA as a result) and other members of the staff whose job is much more delimited and circumscribed to particular tasks and thus send and receive fewer email messages (and have low SA as a result). In other words, it is possible that the pattern of communications reflects a division of labor that emerged among the mission command staff, as their functional role assignments were fixed and relate to their chosen military occupational specialty, an enduring property of their profession.

## Discussion

At a large-scale, 2-week military training event exercise, we conducted a social network analysis of email communications among a multi-echelon mission command staff to assess the commonly held assumption that increased information sharing improves situation awareness among the staff in complex networked operational environments. Results from our exponential random graph models challenge this assumption, as we found that increased email output was associated with lower individual situation awareness. Conversely, higher SA was associated with a lower probability of out-ties, so that sending too many messages broadly to other individuals decreased the likelihood of attaining SA. This challenges our hypothesis that increased information sharing improves situation awareness and also supports a recent laboratory studies that increasing task-relevant information did not improve task performance, but instead reduced self-reported SA, leading to poorer task performance (Marusich et al., 2016). In addition, we observed two strong effects of homophily in email communication. Links were more commonly formed between members of the command staff with similar functions and levels of situation awareness, than between two individuals with dissimilar functions and levels of situation awareness. These findings have major implications that challenge the current conceptual framework of NEO (Alberts and Garstka, 2004) which posits that robust networking and information sharing act as a positive feedback loop resulting in greater situation awareness and mission effectiveness in military operations. These and other results highlight several major growing pains for networked organizations and military organizations in particular.

### Unequal information sharing

The first growing pain for organizations is that information is not shared equally, even in robust and relatively unconstrained information sharing environments. In our observed mission command network, there were large imbalances to information sharing as a few key individuals dominated information sharing among the staff. Most individuals in the network have very few email connections, but a small number of individuals have very many connections. The dominance of key members of the Mission Command staff conforms to a general Pareto-type network property of complex systems (see West, 2012). At network levels of interaction, understanding the social and cognitive dynamics that give rise to Pareto's law constitutes a fundamental question for network science research. Intuitively, it is possible that the degree distribution imbalance occurs whenever there is a fundamental imbalance in the value of individuals in the network. In our mission command network the value of individuals is reflected by military rank/experience and the primacy of certain functional role-positions. If so, this phenomenon could extend beyond military networks to include any workgroup structured using an organizational hierarchy, especially corporations, bureaucracies, departments, and work-groups among others.

In networked organizations, the sheer volume and rapid pace of information and communications received and readily accessible through computer networks can be overwhelming to individuals, resulting in data overload from diverse data sources, multiple data formats, and large data volumes. The need to integrate and interpret information in massive data environments and the macrocognitive processes involved in fashioning a coherent understanding is commonly referred to as sensemaking (Klein et al., 2006). Given the Pareto-type imbalances to the email degree distributions, it is likely that some individuals in the network are beyond their functional cognitive capacity to process and make sense of so much information. It is the case that in complex tasks, limitations in cognitive resources and processes have been shown to give rise to many cognitive biases that distort human decision making (Lebiere et al., 2013). However, humans are remarkably resilient in adapting to the complexity and functional limitations of their environment. Researchers have documented a variety of cognitive strategies and systematically examined the tradeoffs and shortcuts involved in overcoming fixed limits to human information processing capacities, such as attention bottlenecks and memory limitations (see Reitter and Lebiere, 2012). One of those tradeoffs and associated techniques is whether to share raw information, providing all the needed information at the cost of potentially overwhelming attentional demands, or high-level summaries and conclusions, requiring context-sensitive filtering and inference that may miss critical issues in the presence of information stovepipes (Tang et al., 2015).

As a practical consideration, following the business maxim put forward by Koch (2011) in his book, *The 80/20 Principle*, efforts should be made to support this vital 20% that also generates 80% of the work. This suggests that technological solutions and training regimens should focus on supporting the vital 20% of the networked staff driving most of the collaborations (for a decision-support agent approach, see Buchler et al., 2014). The long-tailed distributions of communications have major implications for the psychological and social sciences as many parametric statistical approaches and human performance modeling tools assume some degree of normality in the processes they model (Warwick et al., 2013). As discussed below, understanding how cognition is manifest at network levels of interaction represents a challenge and opportunity for macro-cognitive researchers.

Scientists and engineers have developed many approaches for understanding and predicting individual and group state, behavior, cognition, and performance in the context of teams, organizations, and societies; with each approach being limited in resolution, validity, and insight into the human condition. Understanding how humans interact and adapt within dynamic, complex, natural environments remains a pressing and challenging scientific problem. Recent technological advances have lead researchers and information technology firms (e.g., Navaroli and Smyth, 2015) to leverage vast quantities of data from various human, information, and communication networks to make interpretations and predictions about humans and the context in which they are operating. Network science approaches allow both the organizational context and real-world human behavior to be jointly analyzed and interpreted. However, network science focuses on the interactions between decision-makers and their emergent social phenomena, often oversimplifying many cognitive aspects of the individual nodes. This represents both a challenge and opportunity for macro-cognitive research to define cognitive processes that occurs at the “nodal level” in real-world contexts, such as decision-making under uncertainty and sense-making. In essence, the defining challenge is to understand the cognitive processes that give rise to the heavy-tailed statistics seen at network levels of interaction. For instance, a cognitive mechanism formally implemented at the nodal level as a priority communication model—sorting communication messages by importance in a queue (i.e., email inbox)—was shown in simulation to give rise to the patterns of real-world bursty communication timings observed at the network level (Vázquez et al., 2006).

### Organizational stovepipes

A second growing pain for organizations is one of breaking open “information stovepipes” or existing socio-technical limitations that restrict the free flow of information and communications (e.g., Bateman, 1996). The flow of information among the Mission Command staff involves the timely push and pull of information and knowledge products to and from adjacent, higher, and lower functional cells and units. The distribution of information, however, was largely constrained to and adhered to unit structure of the organization, and thus largely occurred within functional cell assignments. The pattern of communications in our networked organization conform to well-established principles of social networks as we observed strong effects of reciprocity, triadic closure, and within-cell homophily that were governed by their functional cell assignment. These functional cells are well-defined and known according to the general staff system (Department of the Army Headquarters, 2015) and include: command, maneuver, intelligence, fires, civil affairs, signal, sustainment, protection, and liaison. This is the hallmark of a stovepiped organization where information is bottled-up and not widely shared among diverse individuals in the organization. The general pattern of results raise fundamental questions as to the macro-cognitive mechanisms existing at the individual node level that give rise to the patterns observed at the level of the networked organization.

It is not clear how to promote diverse heterophilous ties within an organization. Currently, two theories have been advanced for a lack of heterophilious ties in organizational settings (Chung et al., 2000). First, rank confers status within the Mission Command network and higher-status individuals and organizations in the multi-echelon hierarchy may see their status reduced by ties to lower-status individuals and organizations (Benjamin and Podolny, 1999). In this case, the propensity is to communicate with high-rank individuals. It is possible that this propensity to concentrate communications to high-ranking individuals can drive the types of in-degree imbalances we observed in our email communication network. Indeed, many of the individuals at the tail of the degree distribution are high-ranking principal members of the Division mission command staff. Second, individuals and organizations may have access to unequal information quality which reduces the value proposition of information exchanges between individuals with dissimilar situation awareness. In addition, maintaining heterophilous relationships across functional cells and across unit echelons can be, in practice, quite difficult due to dissimilar work processes, complex information requirements, lack of awareness, and the multitude of disparate information systems that can constrain such collaborations.

### Emergence of information sources and sinks

A third growing pain is the emergence of individuals that function increasingly as sources and sinks of information in the networked organization. From an information flow perspective (Ahlswede et al., 2000), network ties are social channels that allow the flow of information throughout the organization. We observed that by Week 2 of the exercise, with more incoming ties individual members of the Mission Command staff were less likely to send out larger volumes of emails. With more outgoing ties, individuals were also less likely to receive more messages. This suggests that certain individuals increasingly act as sources and sinks in the networked organization and suggests a specialization of information sharing behavior as either broadcasters or attractors of email communications. This also reinforces the primacy of our earlier result that information is not shared equally in the network with Pareto-type imbalances to the in-degree and out-degree distributions. Furthermore, these source and sink effects are emergent properties of the organization. These results support earlier research from military field exercises demonstrating that SA is concentrated to a few select individuals and linked to the participants' awareness of the information in the central nodes of a team (Saner et al., 2009).

### Stratified situation awareness

A fourth growing pain was that over time our organizational network appears to become stratified with respect to SA—an effect of homophily with respect to SA. Those with high situation awareness were likely to have ties to others who also have high SA. Conversely, those who have low SA were likely to have ties to others who also have low SA, and thus have impoverished information flows. The effects of homophily and SA emerged during Week 2 of the military training event exercise and is likely a self-reinforcing phenomenon. This could be the result of deliberate action—individuals with higher SA may reach out to others with high SA while avoiding those with lower SA—or this may be an outcome of the structural configuration of the network—those with access to information that enhances SA were unable to diffuse that information to parts of the network and, as a result, SA declined among those subgroups.

The stratification of SA in this network is problematic for organizational performance and this problem deserves further attention both to advance organizational research and improve the effectiveness of military performance during training exercises. One possibility is that the organization is essentially divided into information processors whose job it is to understand the situation and who receive and send a lot of email messages (and have high SA as a result) and other members of the staff whose job is much more delimited and circumscribed to particular tasks and thus send and receive fewer email messages (and have low SA as a result). In other words, it is possible that the pattern of communications reflect a division of labor that emerged among the mission command staff, as their functional role assignments were fixed and relate to their chosen military occupational specialty, an enduring property of their profession.

Given our results, it is a likely that the stratification of SA emerges as a consequence of the information sharing behavior of the organization to include homophilous ties (and lack of heterophily) and Pareto-type imbalances in the degree distribution. An open question that can be tackled through simulation is whether one or more general mechanisms can produce the observed pattern of results as an emergent process of the organization. That is, it is possible that the generally observed properties of email homophily, reciprocity, and triadic closure can result in Pareto-type imbalances in the degree distribution, which can in turn lead to organizational stovepipes among the staff, sources and sink effects, and ultimately the stratification of situation awareness. Overall, our result suggests that SA is stratified across the networked organization and that a person's role and position within an organization affects and potentially limits the level of shared SA that can be achieved.

Our approach focused on relating individual SA to network levels of interaction among the Mission Command staff. A more nuanced approach for future research involves defining SA in relation to the information requirements required for a given staff role position and unit. Each member of the team provides valuable and critical information within and across roles. For instance, team members in different roles (e.g., commanders, intelligence officers, logistics officers) have common information requirements and also some that are unique to their functional role (Artman and Garbis, 1998). In this case, SA is defined at the aggregate team level and furthermore is also used to define common or overlapping information requirements necessary for shared SA. Although potentially useful to support teammates, it is not necessary for each member of the team to have all the information needed by others on the team. It is important, however, that each team member understands what information is needed to support multiple role positions. Shared SA refers to the degree to which team members have the same SA on a defined set of shared information requirements (Endsley and Jones, 2013). For effective team performance, Team SA refers to the sum total of information and degree to which each team member obtains the SA needed to fulfill his or her responsibilities (Endsley, 1995). It is the case that these are overlapping and mutually defined sets of information that are derived from individual SA.

Many of these challenges faced by our Mission Command staff reflect broad trends and challenges in networked organizations and how to effectively manage the systematic convergence of people, information, and technology in work-directed networked organizations. It is likely that many of the findings that we observed in our Mission Command network are also evident in other organizations.

## Conclusion

The military transformation to NEO has proceeded under a conceptual framework that attempts to exploit the increasing interconnectedness between organizational units to allow more communication, information sharing, cooperation and thereby flexibility, adaptability, and mission effectiveness (Alberts, 2002; Alberts and Hayes, 2003). Our results highlight many challenges (i.e., growing pains) to NEO and the need for fundamental research to guide this transformation; much of the rapidly growing literatures in network science, organizational and team processes, and cognitive science do not fully address many of the presenting problems of complex operational environments, macro-cognition, human-in-the-loop systems, and the defining characteristics of work-driven organizations. The vast majority of insights have been gained through laboratory research using highly controlled contexts and environments. Many of these laboratory studies employ reductive scientific approaches (i.e., divide and conquer) that do not scale to complex real-world operations or larger networks and organizational settings. Recent advances in technology have led researchers and industry to leverage vast quantities of data from various human, information, and communication networks to make interpretations and predictions about humans and the context in which they are operating. Such “big science” approaches are fundamentally multi-disciplinary endeavors involving teams of scientists and engineers that embrace the complexity of real-world phenomena to examine network levels of interaction. Embracing complexity is a key challenge and conceptually is a paradigm-shift for science. Such “big science” approaches will certainly yield fundamental insights and understanding into many complex real-world phenomena, but may not be able to completely predict complex real-world phenomena that are non-deterministic, non-linear, and sensitive to initial conditions and feedback loops (see Arney et al., 2015).

## Author contributions

NB contributed to the ideas, design, execution of the study as well as the analyses of results and write up of the manuscript. SF contributed to data preparation, analyses, particularly social network analyses, and write up of results. LM contributed to data preparation, data analyses, and write up of methods and results. DU contributed to material preparation, data collection, and execution of the study. CL contributed to writing the manuscript, interpreting the results and framing the introduction. CG contributed to writing the manuscript, framing the introduction, and interpreting the results.

## Ethics statement

This study was carried out in compliance of federal and Army Research Laboratory regulations requiring Institutional Review Board review of all research involving human subjects prior to the initiation of a research protocol to ensure the safe and ethical treatment of humans as subjects in research.

### Conflict of interest statement

The authors declare that the research was conducted in the absence of any commercial or financial relationships that could be construed as a potential conflict of interest.

## Appendix

**Table:**

	**Coef**.	**SE**
**ERGM: Week 1**		
Edges	−4.705^***^	0.305
Within-cell homophily	1.200^***^	0.099
Messages sent (in-ties)	−0.001	0.000
Messages sent (out-ties)	0.005^***^	0.000
Messages received (in-ties)	0.004^***^	0.001
Messages received (out-ties)	−0.002^***^	0.001
Messages sent heterophily	−0.002^***^	0.000
Messages received heterophily	−0.002^***^	0.000
SA (in-ties)	0.159	0.384
SA (out-ties)	0.081	0.326
SA heterophily	−0.163	0.309
Reciprocity	2.002^***^	0.153
Triadic closure (GWESP)	0.503^***^	0.050
GWESP alpha	1.498^***^	0.017
GW outdegree	−0.439	0.350
GW indegree	2.352^***^	0.652
	AIC: 3980	BIC: 4092
**ERGM: Week 2**		
Edges	−5.235^***^	0.300
Within-cell homophily	1.105^***^	0.102
Messages sent (in-ties)	−0.004^***^	0.001
Messages sent (out-ties)	0.006^***^	0.000
Messages received (in-ties)	0.007^***^	0.001
Messages received (out-ties)	−0.004^***^	0.001
Messages sent heterophily	−0.001^*^	0.000
Messages received heterophily	−0.003^***^	0.001
SA (in-ties)	1.060^**^	0.395
SA (out-ties)	−0.989^*^	0.341
SA heterophily	−1.351^***^	0.331
Reciprocity	2.080^***^	0.149
Triadic closure (GWESP)	0.750^***^	0.061
GWESP alpha	1.301^***^	0.016
GW outdegree	0.366	0.332
GW indegree	4.535^***^	0.848
	AIC: 4110	BIC: 4223

* *< 0.05*,

** *< 0.01*,

*** *< 0.001*.

## References

[B1] Ahlswede R. Cai N. Li S. Y. R. Yeung R. W. (2000). Network information flow. Inform. Theor. IEEE Trans. 46, 1204–1216. 10.1109/18.850663

[B2] Alberts D. (2002). Information Age Transformation: Getting to a 21st Century Military. Washington, DC: Command and Control Research Program (CCRP) Publications.

[B3] Alberts D. Garstka J. (2004). Network Centric Operations Conceptual Framework Version 2.0. Technical Report, US Office of Force Transformation and Office of the Assistant Secretary of Defense for Networks and Information Integration, US Department of Defense.

[B4] Alberts D. Garstka J. Stein F. (1999). Network Centric Warfare: Developing and Leveraging Information Technology. Washington, DC: CCRP Publication Series, Department of Defense.

[B5] Alberts D. S. Hayes R. E. (2003). Power to the Edge: Command and Control in the Information Age. Washington, DC: Command and Control Research Program (CCRP) Publications.

[B6] Arney C. Koban D. Juliano N. Sobiesk M. (2015). Tomorrow's science connections: networks, measures, motivations, in 23rd ARL USMA Technical Symposium (Aberdeen Proving Ground, MD).

[B7] Artman H. Garbis C. (1998). Team communication and coordination as distributed cognition, in 9th Conference of Cognitive Ergonomics (Limerick), 151–156.

[B8] Barabási A. L. Albert R. Jeong H. (1999). Mean-field theory for scale-free random networks. Phys. A Stat. Mech. Appl. 272, 173–187. 10.1016/S0378-4371(99)00291-5

[B9] Bateman R. L. (1996). Force XXI and the death of *Auftragstaktik*. Armor 1, 13–15.

[B10] Bedny G. Meister D. (1999). Theory of activity and situation awareness. Int. J. Cogn. Ergon. 3, 63–72. 10.1207/s15327566ijce0301_5

[B11] Benjamin B. A. Podolny J. M. (1999). Status, quality, and social order in the California wine industry. Admin. Sci. Q. 44, 563–589. 10.2307/2666962

[B12] Blasch E. Bossé É. Lambert D. A. (2012). High-Level Information Fusion Management and Systems Design. Boston, MA: Artech House.

[B13] Blasch E. P. Breton R. Valin P. (2011). Information fusion measures of effectiveness (MOE) for decision support, in Proc. SPIE 8050, Signal Processing, Sensor Fusion, and Target Recognition XX, 805011, ed Ivan Kadar (Orlando, Fl). 10.1117/12.883988

[B14] Bolstad C. A. Endsley M. R. (2003). Measuring shared and team situation awareness in the army's future objective force, in Proceedings of the Human Factors and Ergonomics Society Annual Meeting, Vol. 47 (Denver, CO: SAGE Publications), 369–373.

[B15] Borgatti S. P. Cross R. (2003). A relational view of information seeking and learning in social networks. Manage. Sci. 49, 432–445. 10.1287/mnsc.49.4.432.14428

[B16] Borgatti S. P. Mehra A. Brass D. J. Labianca G. (2009). Network analysis in the social sciences. Science 323, 892–895. 10.1126/science.1165821 19213908

[B17] Buchler N. Marusich L. R. Sokoloff S. (2014). The Warfighter Associate: decision-support software agent for the management of intelligence, surveillance, and reconnaissance (ISR) assets, in Proceedings of SPIE, Vol. 9079, 907902, International Society for Optics and Photonics, Ground/Air Multisensor Interoperability, Integration, and Networking for Persistent ISR V, SPIE Defense, Security, and Sensing, ed Kolodny M. A. (Baltimore, MD). 10.1117/12.2054646

[B18] Castells M. (2009). The Rise of the Network Society: The Information Age: Economy, Society, and Culture, Vol. 1 Malden, MA: Wiley-Blackwell.

[B19] Chen J. Y. Barnes M. J. Harper-Sciarini M. (2011). Supervisory control of multiple robots: human-performance issues and user-interface design. IEEE Trans. Syst. Man Cybernet. C Appl. Rev. 41, 435–454. 10.1109/TSMCC.2010.2056682

[B20] Chung S. A. Singh H. Lee K. (2000). Complimentarity, status similarity and social capital as drivers of alliance formation. Strateg. Manage. J. 21, 1–22.

[B21] Contractor N. S. Monge P. R. (2002). Managing knowledge networks. Manage. Commun. Q. 16:249 10.1177/089331802237238

[B22] Department of the Army Headquarters (2015). Field Manual 6-0 Commander and Staff Organization and Operations. Washington, DC.

[B23] Department of Homeland Security (2013). DHS Information Sharing and Safeguarding Strategy. Available online at: http://www.dhs.gov/sites/default/files/publications/12-4466-dhs-information-sharing-and-safeguarding-strategy-01-30-13–fina%20%20%20.pdf

[B24] Endsley M. R. (1995). Toward a theory of situation awareness in dynamic systems. Hum. Factors J. Hum. Factors Ergon. Soc. 37, 32–64. 10.1518/001872095779049543

[B25] Endsley M. R. (2000). Theoretical underpinnings of situation awareness: a critical review, in Situation Awareness Analysis and Measurement. eds Endsley M. R. Garland D. J. (Mahwah, NJ: Lawrence Erlbaum Associates), 3–32.

[B26] Endsley M. R. Jones W. (2013). Situation awareness, in The Oxford Handbook of Cognitive Engineering, eds Lee J. D. Alex Kirlik (New York, NY: Oxford University Press), 88–108.

[B27] Endsley M. R. Kiris E. O. (1995). Situation Awareness Global Assessment Technique (SAGAT) TRACON Air Traffic Control Version User Guide. Lubbock, TX: Texas Tech University.

[B28] Endsley M. R. Smolensky M. W. (1998). Situation awareness in air traffic control: the picture, in Human Factors in Air Traffic Control, eds Smolensky M. W. Stein E. S. (San Diego, CA: Academic Press), 115–154.

[B29] Federal Aviation Administration (2014). NextGen Implementation Plan. Washington, DC.

[B30] Gleick J. (2012). The Information: A History, a Theory, a Flood. New York, NY: Random House LLC.

[B31] Gonzalez C. Wimisberg J. (2007). Situation awareness in dynamic decision making: effects of practice and working memory. J. Cogn. Eng. Decis. Making 1, 56–74. 10.1177/155534340700100103

[B32] Hackman J. R. Katz N. (2010). Group behavior and performance. Handb. Soc. Psychol. 3:32, 1208–1251. 10.1002/9780470561119.socpsy002032

[B33] Handcock M. Hunter D. Butts C. Goodreau S. Krivitsky P. Morris M. (2016). ergm: Fit, Simulate and Diagnose Exponential-Family Models for Networks. The Statnet Project (http://www.statnet.org). R package version 3.6.0. Available online at: http://CRAN.R-project.org/package = ergm

[B34] Hauss Y. Eyferth K. (2003). Securing future ATM-concepts' safety by measuring situation awareness in ATC. Aerosp. Sci. Technol. 7, 417–427. 10.1016/S1270-9638(02)00011-1

[B35] Hunter D. R. (2007). Curved exponential family models for social networks. Soc. Networks 29, 216–230. 10.1016/j.socnet.2006.08.005 18311321 PMC2031865

[B36] Hunter D. R. Handcock M. S. (2006). Inference in curved exponential family models for networks. J. Comput. Graph. Stat. 15, 565–583 10.1198/106186006X133069

[B37] Hunter D. R. Handcock M. S. Butts C. T. Goodreau S. M. Morris M. (2008). ergm: A package to fit, simulate and diagnose exponential-family models for networks. J. Stat. Softw. 24:nihpa54860. 10.18637/jss.v024.i03 19756229 PMC2743438

[B38] Jacomy M. (2009). Force-Atlas Graph Layout Algorithm. Available online at: https://gephi.org/tutorials/gephi-tutorial-layouts.pdf

[B39] Kaber D. B. Endsley M. R. Wright M. C. Warren H. (2002). The Effects of Levels of Automation on Performance, Situation Awareness, and Workload in An Advanced Commercial Aircraft Flight Simulation. Final Report: NASA Langley Research Center Grant# NAG-1-01002, Hampton, VA: NASA Langley Research Center.

[B40] Keller J. Lebiere C. Shay C. R. Latorella K. (2004). Cockpit system situational awareness modeling tool, in Human Performance, Situation Awareness and Automation: Current Research and Trends: HPSAA II, Vol. 2, eds Vincenzi D. A. Mouloua M. Hancock P. A. (Daytona Beach, FL: Psychology Press), 66–71.

[B41] Klein L. A. (2004). Sensor and Data Fusion: A Tool For Information Assessment and Decision Making, Vol. 324 Bellingham, WA: SPIE Press.

[B43] Klein G. Moon B. Hoffman R. R. (2006). Making sense of sensemaking 2: a macrocognitive model. IEEE Intell. Syst. 21, 88–92. 10.1109/MIS.2006.100

[B44] Klein G. Ross K. G. Moon B. M. Klein D. E. Hoffman R. R. Hollnagel E. (2003). Macrocognition. IEEE Intell. Syst. 18, 81–85. 10.1109/MIS.2003.1200735

[B45] Koch R. (2011). The 80/20 Principle: The Secret to Achieving More with Less. New York, NY: Crown Business.

[B46] Kozlowski S. W. Gully S. M. Nason E. R. Smith E. M. (1999). Developing adaptive teams: a theory of compilation and performance across levels and time, in The Changing Nature of Performance: Implications for Staffing, Motivation, and Development, ed Pulakos (San Francisco, CA: Jossey-Bass Publishers), 240–292.

[B47] Lebiere C. Pirolli P. Thomson R. Paik J. Rutledge-Taylor M. Staszewski J.. (2013). A functional model of sensemaking in a neurocognitive architecture. Comput. Intell. Neurosci. 2013:921695. 10.1155/2013/921695 24302930 PMC3835765

[B48] Marusich L. M. Buchler N. (2016). Time series modeling of Army mission command communication networks: an event-driven analysis. J. Comput. Math. Organ. Theor. 10.1007/s10588-016-9211-7 Available online at: http://link.springer.com/article/10.1007/s10588-016-9211-7

[B49] Marusich L. R. Bakdash J. Z. Onal E. Michael S. Y. Schaffer J. O'Donovan J.. (2016). Effects of information availability on command-and-control decision making performance, trust, and situation awareness. Hum. Factors 58, 301–321. 10.1177/0018720815619515 26822796

[B50] Matthews M. D. Strater L. D. Endsley M. R. (2004). Situation awareness requirements for infantry platoon leaders. Mil. Psychol. 16, 149 10.1207/s15327876mp1603_1

[B51] McGrath J. E. Kravitz D. A. (1982). Group research. Annu. Rev. Psychol. 33, 195–230. 10.1146/annurev.ps.33.020182.00121126573069

[B52] McPherson M. Smith-Lovin L. Cook J. M. (2001). Birds of a feather: homophily in social networks. Annu. Rev. Sociol. 27, 415–444. 10.1146/annurev.soc.27.1.415

[B53] Navaroli N. Smyth P. (2015). Modeling response time in digital human communication, in Ninth International AAAI Conference on Web and Social Media. Oxford, UK.

[B54] Pareto V. (1897). Cours d'Economie Politique. Lausanne; Paris.

[B55] Patrick J. James N. Ahmed A. (2007). Awareness of Control Room Teams. Paris: Presses Universitaires de France.

[B56] Reitter D. Lebiere C. (2012). Social cognition: memory decay and adaptive information filtering for robust information maintenance, in Proceedings of the Twenty-Sixth AAAI Conference on Artificial Intelligence (AAAI-12). Toronto, ON.

[B57] Roby T. B. Lanzetta J. T. (1958). Considerations in the analysis of group tasks. Psychol. Bull. 55:88. 10.1037/h0047233 13527594

[B58] Salas E. Goodwin G. F. Burke C. S. (2008). Team Effectiveness in Complex Organizations: Cross-Disciplinary Perspectives and Approaches. New York, NY: Routledge.

[B59] Salimi N. Vita R. (2006). The biocurator: connecting and enhancing scientific data. PLoS Comput. Biol. 2:e125. 10.1371/journal.pcbi.0020125 17069454 PMC1626147

[B60] Salmon P. Stanton N. Walker G. Green D. (2006). Situation awareness measurement: a review of applicability for C4i environments. Appl. Ergon. 37, 225–238. 10.1016/j.apergo.2005.02.001 16023612

[B61] Saner L. D. Bolstad C. A. Gonzalez C. Cuevas H. M. (2009). Measuring and predicting shared situation awareness in teams. J. Cogn. Eng. Decis. Making 3, 280–308. 10.1518/155534309X474497

[B62] Scott J. (2012). Social Network Analysis. Los Angeles, CA: Sage.

[B63] Snijders C. Matzat U. Reips U. (2012). Big data”: big gaps of knowledge in the field of internet science. Int. J. Internet Sci. 7, 1–5.

[B64] Smith K. Hancock P. A. (1995). Situation awareness is adaptive, externally directed consciousness. Hum. Factors 37, 137–148.

[B65] Sonnenwald D. H. Pierce L. G. (2000). Information behavior in dynamic group work contexts: interwoven situational awareness, dense social networks and contested collaboration in command and control. Inform. Process. Manage. 36, 461–479. 10.1016/S0306-4573(99)00039-4

[B66] Strogatz S. H. (2001). Exploring complex networks. Nature 410, 268–276. 10.1038/35065725 11258382

[B67] Swan J. M. Hennig J. (2012). From Data to Decisions. Army Acquisition, Logistics, and Technology (AL and T), 8–12.

[B68] Tang Y. Lebiere C. Sycara K. Morrison D. Lewis M. Smart P. (2015). Information sharing for collective sensemaking, in Proceedings of the Hawaii International Conference on System Sciences (HICSS-49) (Koloa, HI: IEEE Digital Library).

[B69] Taylor R. M. (1990). Situation awareness rating technique (SART): the development of a tool for aircrew systems design, in Proceedings of the AGARD AMP Symposium on Situation Awareness in Aerospace Operations (Neuilly-sur-Seine: Advisory Group for Aerospace Research and Development), 3-1–3-17.

[B70] Warwick W. Buchler N. Marusich L. (2013). Network science and human performance modeling, in Proceedings of the Human Factors and Ergonomic Society (San Diego, CA).

[B71] West B. J. (2012). Complex Worlds: Uncertain Unequal and Unfair. Castroville, TX: Black Rose Writing.

[B72] White T. (2012). Hadoop: The Definitive Guide, 3rd Edn. Sebastopol, CA: O'Reilly Media, Inc.

[B73] Wright M. C. Taekman J. M. Endsley M. R. (2004). Objective measures of situation awareness in a simulated medical environment. Qual. Safe. Health Care 13, i65–i71. 10.1136/qshc.2004.009951 15465958 PMC1765787

[B74] Vázquez A. Oliveira J. G. Dezsö Z. Goh K. I. Kondor I. Barabási A. L. (2006). Modeling bursts and heavy tails in human dynamics. Phys. Rev. E 73:036127. 10.1103/PhysRevE.73.036127 16605618

[B75] Zachary W. W. (1977). An information flow model for conflict and fission in small groups. J. Anthropol. Res. 33, 452–473. 10.1086/jar.33.4.3629752

[B76] Zheng X. S. Tai Y. C. McConkie G. W. (2004). Exploring drivers' situation awareness in a dynamic traffic environment, in Proceedings of the Human Factors and Ergonomics Society Annual Meeting. New Orleans, LA: SAGE Publications.

